# Cholestasis in Benign Recurrent Intrahepatic Cholestasis 2

**DOI:** 10.14309/crj.0000000000000412

**Published:** 2020-06-22

**Authors:** Eric Arthur Lorio, David Valadez, Naim Alkhouri, Nicole Loo

**Affiliations:** 1 Department of Medicine, University of Texas Health, San Antonio, TX; 2 Division of Gastroenterology, Texas Liver Institute, University of Texas Health, San Antonio, TX

## Abstract

Benign recurrent intrahepatic cholestasis represents a rare class of autosomal recessive chronic cholestasis disorders, usually presenting with recurrent episodes of intense pruritus and jaundice. We report a 27-year-old woman presenting with benign recurrent intrahepatic cholestasis type 2 due to heterozygosity in *ABCB11.* Interestingly, she was also found to be heterozygous in cystic fibrosis transmembrane conductance regulator, *NPHP4*, and *A1ATD* (*SERPINA1*), which may explain the severe nature of her disease expression because heterozygosity in each of these genes has been associated with cholestasis. Finally, she exhibited a response to steroids that may have implications for future treatment of bile salt export pump-related diseases.

## INTRODUCTION

Benign recurrent intrahepatic cholestasis (BRIC) represents a class of autosomal recessive disorders characterized by intermittent bouts of cholestasis.^1^ BRIC type 1 results from mutations in *ATP8B1*, whereas BRIC type 2 is caused by heterozygous mutations in the *ABCB11* gene (2q24) encoding the ATP-dependent bile salt export pump (BSEP) protein in intrahepatic biliary ducts. Recurrent cholestasis and jaundice are hallmarks of the disease. By definition, BRIC does not progress to advanced liver disease, although repeat episodes can contribute to a marked reduction in quality of life, and any BSEP deficiency increases the risk of hepatobiliary malignancy (estimated 15% lifetime risk).^2^ One of the diagnostic challenges of BRIC is that aminotransferase activity can be normal during cholestatic attacks, and liver biopsies often show nonspecific hepatocanalicular cholestasis. BRIC diagnosis requires a high degree of clinical suspicion and can only be confirmed with genetic testing for corresponding mutations in *ATP8B1* and *ABCB11*. We present a patient with multiple hospitalizations for debilitating fatigue, weakness, and pruritus, and we demonstrate a BRIC-2 response to corticosteroids that may represent a novel therapy for treatment-refractory disease.

## CASE REPORT

A 27-year-old Hispanic woman presented with jaundice, pruritus, and weakness for 4 weeks. This marked her third similar admission, having first presented with similar symptoms 3 years earlier when she was pregnant with her first child. At that time, she had a total bilirubin (TB) level of 13.2 and was diagnosed at 22 weeks with intrahepatic cholestasis of pregnancy (ICP). Hepatitis A, B, C, antinuclear antibody, antismooth muscle antibody, anti-liver/kidney microsome-1 antibody, antimitochondrial antibody, ceruloplasmin, ferritin, iron saturation, α-1 antitrypsin, right upper quadrant ultrasound, and magnetic resonance cholangiopancreatography were all negative, and signs and symptoms resolved after an uncomplicated preterm cesarean section.

She suffered symptom recurrence 8 months later, exhibiting jaundice, pruritus, and increasing fatigue and weakness. Elevated TB, direct bilirubin (DB), alkaline phosphatase, and transaminases were noted, with a negative urine β-human chorionic gonadotropin. Abdominal magnetic resonance imaging showed absent cholelithiasis, choledocholithiasis, or biliary obstruction. Liver biopsy showed histologic features of nonspecific cholestasis, suggesting extrahepatic duct obstruction. Her hepatic enzymes and symptoms gradually improved with supportive treatment, and she was discharged with a diagnosis of unresolved ICP.

On this presentation 2 years after her second discharge, she reported 4 weeks of progressively worsening jaundice, pruritus, weakness, and fatigue. As with previous admissions, the initial approach consisted of supportive treatment with ursodeoxycolic acid (UDCA), cholestyramine, camphor/menthol lotion, tramadol, and dicyclomine. Aspartate transaminase and alanine transaminase were 63 and 74 U/L, respectively, and she was found to have a TB of 9.1 μmol/L, DB of 6.7 μmol/L, GGT of 22 U/L, alkaline phosphatase of 227 IU/L, negative hepatitis serology, and a negative β-human chorionic gonadotropin.

**Figure 1.**
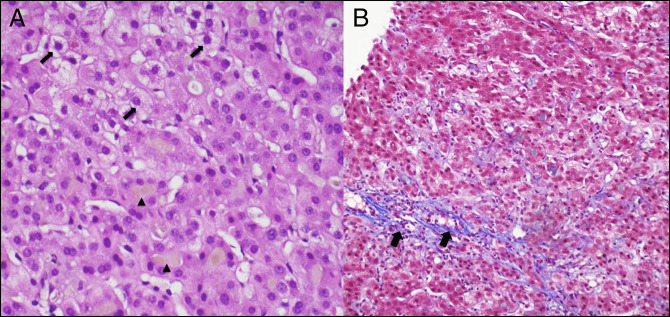
Liver biopsy showing (A) feathery degeneration (arrows), a phenomenon occurring in periportal hepatocytes because of the detergent-like action of bile acids in intrahepatic cholestasis. Arrowheads demarcate bile deposition in the setting of bile stasis and (B) trichrome stain from hepatic biopsy showing the mild hepatocyte pericentral and perisinusoidal fibrosis (arrows) associated with benign recurrent intrahepatic cholestasis. Although fibrosis is the hallmark of progressive familial intrahepatic cholestasis, these diseases likely represent a continuum of disease.

Ultrasound demonstrated a 4-mm common bile duct without gallstones. Magnetic resonance cholangiopancreatography revealed no biliary stricture or dilation, with mild liver enlargement to 19 cm. TB remained elevated at 9.7 μmol/L on day 6, and liver biopsy was performed again. Pathology showed extensive canalicular cholestasis, with associated hepatocyte pericentral and perisinusoidal fibrosis (Figure 1). Despite minimal symptom improvement on day 9 of hospitalization, she provided a blood sample for genetic testing and was discharged home with UDCA and hepatology follow-up.

She returned 2 days later with worsening fatigue, abdominal discomfort, pruritus, and jaundice despite minimal liver enzyme elevation. Supportive care was resumed, and she refused a third liver biopsy as TB and DB peaked at 14.2 and 11.5 μmol/L, respectively, and her symptoms advanced. On day 18 of her third admission, she was started on 40 mg daily IV methylprednisolone. She experienced modest symptomatic improvement with a decrease in TB to 8.1 μmol/L. Twenty-one days after initially presenting, the patient was discharged home with a short prescription for oral methylprednisolone 40 mg daily, having not yet received a definitive diagnosis.

One month later, genetic testing returned positive for heterozygosity in *ABCB11*, a genetic variant causing BRIC type 2 (Table 1). Interestingly, she was also heterozygous in the cystic fibrosis transmembrane conductance regulator gene, which has been implicated in progressive familial intrahepatic cholestasis (PFIC) type 1, and other well-known cholestatic contributors, *NPHP4* and *A1ATD* (*SERPINA1*).

**Table 1.**
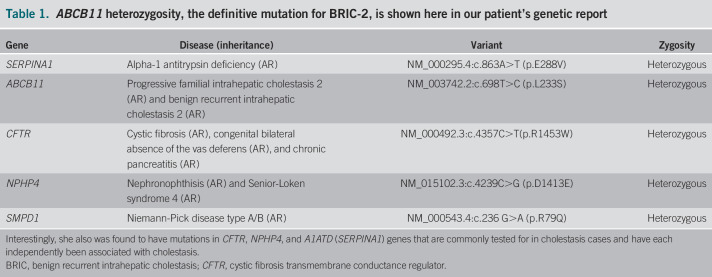
*ABCB11* heterozygosity, the definitive mutation for BRIC-2, is shown here in our patient's genetic report

## DISCUSSION

We present a diagnosis of BRIC that remained elusive through multiple hospitalizations. Liver biopsy revealed hepatocyte pericentral and perisinusoidal fibrosis, which is more suggestive of PFIC, a progressive, fibrotic liver disease due to a homozygous *ABCB11* mutation resulting in at least a 70% reduction in bile acid secretion.^3^ Although BRIC rarely progresses to PFIC, these disorders may not be entirely distinct, and evidence increasingly suggests they may represent a continuum of disease.^4^

Both BRIC2-2 and PFIC2 result from mutations in *ABCB11*, and in vitro studies have demonstrated that residual transport function of mutant BSEP proteins correlates with the phenotypic differences between these 2 diseases.^5^ *ABCB11* mutations can be inherited or de novo, and compound heterozygosity can play a major role in expression, with up to 30% of these patients having heterozygous allele mutations adjacent to *ABCB11.*^6^ Combinations of autosomal recessive allele mutations and additional variable mutations, in this case in *CFTR*, *NPHP4*, and *A1ATD*, likely result in nonsense and frameshift mutations allowing further reduction in residual BSEP function.^6,7^ Immunostaining previously served as the primary means of diagnosis, although with the onset of genetic testing, both factors are considered and the decision to screen family members should be taken into account on a case-by-case basis.^8^

Our patient's response to methylprednisolone offers a potentially novel approach for the treatment of BRIC-2. Current medical therapies hinge on the use of rifampicin, which can reduce pruritus, and UDCA, which is rarely effective.^4^ Steroids have been explored in drug-induced cholestasis, chronic cholestasis secondary to hepatitis, and ICP.^9–12^ However, only recently has a role for steroids been demonstrated in the treatment of BSEP disorders. In 2015, 2 patients with PFIC2, both compound heterozygotes for *ABCB11*, were reported to have achieved significant symptom improvement with normalization of liver enzymes and bile salt levels after starting budesonide for unrelated conditions.^13^ Our case represents a similar, if less dramatic, steroid response and may emphasize the need for further consideration of steroid therapy in the management of BSEP deficiencies.

## Disclosure

Author contributions: All authors contributed equally to this manuscript. E. Lorio is the article guarantor.

Financial disclosure: None to report.

Informed consent was obtained for this case report.

Previous presentation: This case was presented at the American College of Gastroenterology Annual Scientific Meeting; October 25-30, 2019; San Antonio, Texas.
